# Improvements of nuclease and nickase gene modification techniques for the treatment of genetic diseases

**DOI:** 10.3389/fgeed.2022.892769

**Published:** 2022-07-26

**Authors:** Yaoyao Lu, Cedric Happi Mbakam, Bo Song, Eli Bendavid, Jacques-P. Tremblay

**Affiliations:** ^1^ CHU de Québec Research Center, Laval University, Quebec City, QC, Canada; ^2^ Department of Molecular Medicine, Laval University, Quebec City, QC, Canada

**Keywords:** gene editing, ZFN, TALEN, CRISPR-cas, Cytidine Base Editor (CBE), Adenosine Base Editor (ABE), prime editing

## Abstract

Advancements in genome editing make possible to exploit the functions of enzymes for efficient DNA modifications with tremendous potential to treat human genetic diseases. Several nuclease genome editing strategies including Meganucleases (MNs), Zinc Finger Nucleases (ZFNs), Transcription Activator-like Effector Nucleases (TALENs) and Clustered Regularly Interspaced Short Palindromic Repeats-CRISPR associated proteins (CRISPR-Cas) have been developed for the correction of genetic mutations. CRISPR-Cas has further been engineered to create nickase genome editing tools including Base editors and Prime editors with much precision and efficacy. In this review, we summarized recent improvements in nuclease and nickase genome editing approaches for the treatment of genetic diseases. We also highlighted some limitations for the translation of these approaches into clinical applications.

## 1 Introduction

Mutations of a single or several nucleotides in human genome are responsible for major hereditary health problems (Benusiglio et al., 2021; Samuelson et al., 2021; Xiao et al., 2021; Chen et al., 2022; Cobo et al., 2022). To date, about 7,000 hereditary diseases are estimated to be caused by monogenic mutations (Claussnitzer et al., 2020). Homologous recombination (HR) has long been proposed as an avenue to treat human genetic diseases and its efficiency can be increased by inducing the DNA double-strand breaks (DSBs) (Mao et al., 2008). A significant step forward in gene therapy has been the discovery of enzymes known as nucleases. These enzymes enable gene editing technologies to modify a specific DNA sequence within the natural cell environment for the correction of hereditary diseases (Durai, 2005; Cermak et al., 2011; Hwang et al., 2013).

Over the past decades, significant technology development have empowered bio-engineers with tools such as nuclease-mediated Meganucleases (MNs) (Epinat et al., 2003), Zinc Finger Nucleases (ZFNs) (Porteus and Baltimore, 2003; Miller et al., 2007), Transcription Activator-Like Effector Nucleases (TALENs) (Method of the Year 2011, 2012) and Clustered Regularly Interspaced Short Palindromic Repeats-CRISPR associated protein 9 (CRISPR-Cas9) (Jinek et al., 2012a; Ran et al., 2013b; Shalem et al., 2014b). These technologies grant us access to the genome for an accurate base-to-base modification without DSBs by deploying techniques such as Base editing and Prime editing (Gaudelli, 2017; Komor et al., 2017; Anzalone et al., 2019). Theoretically, these gene editing technology enable the replacement of single or multiple bases in any gene of interest at any given location. However, the gene editing effectiveness are influenced by at least three factors: (i) The type of gene editing (e.g., DNA base pair conversion, deletion, insertion, or a combination of the three above changes), (ii) the availability of a gene editing technology to achieve its desired outcome, (iii) the efficiency of the gene editing process. More importantly, the main bottleneck, the acquisition of tissue-specific edits and unwanted genome modification events, is still remains.

To explore how to edit the genome, in this review, we particularly focused on the mechanisms, the limitations and optimizations of six high-profile gene editing technologies as well as the recent progress of various types of genome editing tools used in clinical or preclinical research.

## 2 Nuclease-based genome engineering technologies

**FIGURE 1:**
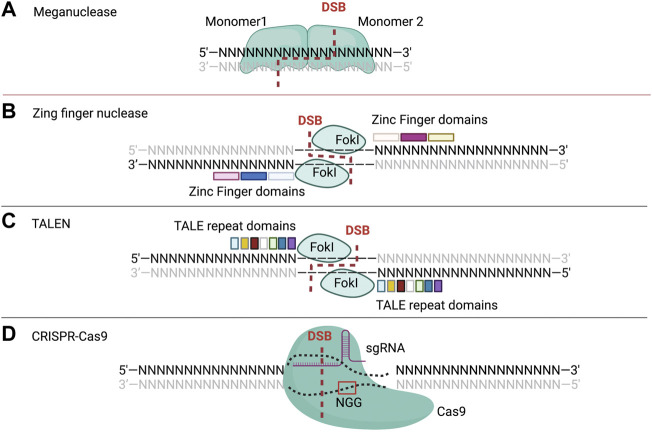
The **(A)** is depicting the two monomer domains (monomer 1 and monomer 2) of mega nuclease which cleave at the binding sites, resulting in the DNA double strand break. The **(B)** shows the two zinc finger domains of ZFNs made of three ZF motifs distinct binding sites (sequence of 3 nucleotides) and cleaving domains (FokI). The **(C)** represents TALE repeat domains (shown in colored squares) pair with distinct binding DNA nucleotides and cleaving domains (FokI). The **(D)** shows the single guide RNA complexed with Cas9 to open and cleave the DNA sequence, trough the recognition of the PAM sequence (by Cas9) which is NGG for SpCas9, and the target sequence (by sgRNA, single guide RNA). N represents any nucleotide amongst A, T, C, and G nucleotide.

In the past decade, various nuclease gene editing technologies have been developed and widely used. These technologies empowered scientists to modify specific sequences in the genome of diverse organisms (Tzfira et al., 2003; Urnov et al., 2010). The most common nucleases-mediated gene editing technologies (Table 1) are MNs (Figure 1A), ZFNs (Figure 1B), TALENs (Figure 1C), and CRISPR-Cas9 (Figure 1D). These technologies combined specific DNA target recognition sequences and programmable endonucleases to induce the desired genomic DNA sequence alterations by introduction of DNA DSB resulting in insertions, deletions, gene replacements and nucleotide substitutions (Lo et al., 2013; Miyaoka et al., 2016). In eukaryotic cells, double-strand DNA cleavage by nucleases triggers two major DNA repair mechanisms including: (i) The non-homologous end joining (NHEJ) and microhomology-mediated end joining through re-ligation of the ends pathways, and (ii) the homology-directed repair (HDR) generated by repairing through a separate donor DNA template (Ranjha et al., 2018). Genome editing takes advantage of these DNA repair processes to produce desired genomic alteration in cell cultures and organisms. (Valerie and Povirk, 2003; Iliakis et al., 2004). However, the DNA DSBs can cause undesired outcomes such as insertions and deletions (Indels) as well as p53 activation (Naeem et al., 2020; Ihry et al., 2018).

**TABLE 1:** The comparison of nuclease-mediated technologies.

	Mega-nuclease	ZFN	TALEN	CRISPR/Cas9
Enzyme	endonuclease	Fok1-nuclease	Fok1-nuclease	Cas9 nuclease
Target site	LAGLIDADG proteins	Zinc-finger binding sites	RVD tandem repeat region of TALE protein	PAM/spacer sequence
Recognition sequence size	12–45 bp	9–18 bp	14–20 bp	3–8 bp/20 bp
Targeting limitations	MN cleaving site	Difficult to target non-G-rich sites	5ʹ targeted base must be a T for each TALEN monomer	Targeted site must precede a PAM sequence
Advantage	1) High specificity	1) Small protein size	1) High specificity	1) Easy to engineer
	2) Relatively easy to deliver *in vivo*	2) Relatively easy *in vivo* delivery	2) Relatively easy to engineer	2) Easy to multiplex
Disadvantage	1) Complex to engineer	1) Expensive	1) Difficult to multiplex	1) Lower specificity
	2) Difficult to multiplex	2)Time-consuming	2) Not applicable for methylcytosine DNA	2) Limited *in vivo* delivery
	3) The target loci need to be engineered into genome	3) Difficult to select the target sequence	3) Limited *in vivo* delivery	
		4) All the ZF domains should be active	4) All the TALEs should be active	

### 2.1 Meganucleases

MNs also referred to as homing endonucleases recognize a large DNA stretches (12–40 base pairs) to facilitate cleavage in the most genomes (Rouet et al., 1994b). MNs are generally encoded by introns or inteins to promote homing of their respective genetic elements into intron or intein-free homologous allelic sites (Epinat et al., 2003). One of the distinctive features of these MNs is their high specificity, due to the tight coupling of their binding site. This tight coupling recognizes a single locus within the yeast nuclear or mitochondrial genome (Paques and Duchateau, 2007). The LAGLIDADG proteins are the most well-studied homing endonuclease. They interact with their targets by nonspecific interactions between the ß strands and the backbone of the target DNA through the recognition of a sequence of 2–4 bp region. Consequently, several new engineered endonuclease variations derived from the following homing endonuclease: (i) I-CreI which was discovered in the chloroplast genome of *Chlamydomonas reinhardtii*, and (ii) I-SceI which is present in the mitochondria of *Saccharomyces cerevisiae* (Prieto et al., 2007; Gao et al., 2010; Zekonyte et al., 2021; Lee et al., 2022). These engineered endonucleases enable *in vivo* and *in vitro* genetic modifications. Due to the tiny molecular weight of modified MNs makes the *in vivo* delivery possible. However, the editing efficiency of this strategy is low compared to later developed nuclease-mediated technologies (Aubert et al., 2016; Zhu et al., 2017). Furthermore, reengineering MNs to expand the spectrum of DNA target sequences is complex and laborious and therefore vastly limit its application (Gouble et al., 2006). Recently, Zekonyte et al. (2021) used the engineered I-CreI meganuclease administered *via* intravenous (IV) injection of AAV9 into mice for the correction of m.5024C>T mutation in the mt-tRNA^Ala^ gene, as a curative method for disorders caused by heteroplasmic mitochondrial DNA mutation. This resulted to the elimination of mutant mitochondrial DNA followed by the restoration of mt-tRNA^Ala^ level. Moreover, this approach has also been used in pig model of autosomal dominant Retinitis Pigmentosa (adRP) for the correction of P23H mutation in the rhodopsin (RHO) gene (Jalligampala et al., 2021) and in non-human primates for the modification of the proprotein convertase subtilisin/kexin type 9 (PCSK9) gene responsible for hypercholesterolemia (Wang et al., 2018a; Wang et al., 2021).

#### 2.1.1 Meganuclease related limitations and perspectives

Since the introduction of MNs application, unexpected drawbacks are constantly being discovered. Some of these challenges are: (i) The targeted locus must contain a specific MN cleavage site for each endonuclease whereas the microbial self-splicing intervening sequence could specifically duplicate into recipient alleles of their host gene lacking such sequence (Rouet et al., 1994a; Stoddard, 2011), (ii) low efficacy (Chapdelaine et al., 2010), and (iii) potential genotoxicity (Suzuki et al., 2020). The natural repertory of homing endonucleases is limited to a finite number of proteins, most of them still being hypothetical or uncharacterized. Thus, for other protein, the cleavage site for each meganuclease has to be inserted into the target genome. Because of this flaw, the application of this technology is significantly limited. However, some engineered enzymes originated from meganuclease I-CreI (Arnould et al., 2011), I-SceI (Siegl et al., 2010) and I-DmoI (a monomeric meganuclease from the hyperthermophilic archaeon *Desulfurococcus mobilis*) (Molina et al., 2016) capable of cleaving DNA in specific genomic sites have been generated. I-CreI plays a critical role in the localization and occupancy of the catalytic metal ions, which is crucial for the DNA cleavage (Prieto et al., 2018). Wang et al. (2022) developed a transgenic Xenopus tropicalis line which is used for evaluating the potential effects of I-SceI mediated transgenesis and further understanding its mechanisms. The fusion of transcription activator-like effector (TALE) DNA-binding domains to MNs dramatically increases the efficiency by 35-fold compared to standalone MNs to modify T-cells receptor alpha (Boissel et al., 2014a). Furthermore, due to specific sites targeted by high cleavage specificity and the long length of the sequences (Petersen and Niemann, 2015b; Izmiryan et al., 2016), low off-target effects were detected in MNs because of the structure of meganucleases and the delivery methods. Some strategies could reduce the off-target by combining meganuclease with TALarray or I-TevI (a GIY-YIG enzyme) (Boissel et al., 2014b; Wang et al., 2018b).

### 2.2 Zinc finger nucleases

Zinc Finger Proteins (ZFPs) are artificially synthetic engineered hybrid heterodimeric proteins for site specific genome editing. ZFPs include a sequence of 3–6 peptides (called Zinc Finger (ZF) domains), each binding to a specific sequence of 3–6 base pairs for a specific attachment to a gene sequence (Durai et al., 2005). Two ZFPs are required to fuse with *Flavobacterium okeanokoites* endonuclease I (FokI) to induce a DSB at a specific genomic site. ZFNs are frequently used for gene silencing and knockout (Santiago et al., 2008; Gutschner et al., 2011; Gaj et al., 2012; Sun et al., 2018). Therefore, ZFNs have emerged as a versatile tool for gene targeting in various mammalian cells and organisms for the treatment of hereditary diseases (Almeida and Matos, 2019) and creation of animal models for diseases (Petersen and Niemann, 2015a).

ZFNs represent the first gene editing method applied for clinical treatment of diseases. ZFNs have been used to modify autologous CD4^+^ T-cells to inhibit the function of the human chemokine (C-C motif) receptor 5 gene (CCR5) receptor and reduce the infection of these cells by HIV (Tebas et al., 2014). The results showed that infusion of genetically modified CD4^+^ T-cells was well tolerated, and the HIV viral load has been decreased in blood level of most patients. Another experiment also showed that the HIV-specific CD8^+^ T-cell responses are substantially restored (Tebas et al., 2021). The ZFN approach was also successfully used for the treatment of ß-hemoglobinopathies (Hoban et al., 2015; Chang et al., 2017; Psatha et al., 2018; Smith et al., 2019). Indeed, the phase I/II clinical trial (NCT03432364) sponsored by Sangamo Therapeutics Inc., aimed to assess the safety, tolerability and efficacy of ST-400 for the treatment of transfusion-dependent beta-thalassemia. The ST-400 are patient’s hematopoietic stem cells genetically modified by ZFNs to disrupt a specific and precise sequence of the enhancer of the BCL11A gene in order to boost the expression of fetal hemoglobin (HbF) (Bauer and Orkin, 2015; Masuda et al., 2016). A cohort of six participants will be completed by November 2022. In other studies involving two patients manifesting different genotypic profiles showed a prompt hematopoietic reconstitution with long term increased HbF levels; however, serious adverse events (e.g., Hypersensitivity) have been recorded with one patient as a result of reengineered ST-400 (Smith et al., 2019). Scientists at Bioverative Inc., a Sanofi company, are conducting a phase I/II clinical trials (NCT03653247) in a cohort of eight patients to evaluate the safety, tolerability and efficacy of autologous hematopoietic stem cell transplantation using BIVV003 for the treatment of severe Sickle Cell Disease (SCD) in adults. This trial was supported by encouraging preclinical results that showed a robust long-term engraftment of *ex-vivo* modified hematopoietic stem and progenitor cells (HSPC) from patients. In other phase I/II clinical trials conducted by Sangamo Therapeutics Inc., the UCSF Benioff Children’s Hospital delivered SB-318 (NCT02702115) and SB-913 (NCT03041324) into participants to insert the corrected copy of α-L-iduronidase (IDUA) and iduronate-2-sulfatase (IDS) transgenes respectively into the albumin locus to provide permanent liver specific expression of iduronidase. Preliminary results showed the evidence of albumin-IDS mRNA transcripts in liver and the hepatocytes are able to generate active IDS enzyme (Muenzer et al., 2019). The summary of ongoing clinical trials is presented in Table 2.

**TABLE 2:** Summary of ZFNs ongoing clinical trials.

Disease	Trial number	Sponsor	Status	Drug	Phase	Completion date
Mucopolysaccharidosis II	NCT03041324	Sangamo Therapeutics	Terminated	SB-913	I/II	May-2021
Mucopolysaccharidosis I	NCT02702115	Sangamo Therapeutics	Terminated	SB-318	I/II	Nov-2021
Hemophilia B	NCT02695160	Sangamo Therapeutics	Terminated	SB-FIX	I	Apr-2021
Beta-thalassemia	NCT03432364	Sangamo Therapeutics	Active	ST-400	I/II	Nov-2022
Mucopolysaccharidosis, Hemophilia	NCT04628871	Sangamo Therapeutics	Enrolling	SB-913, SB-318, SB-FIX	NA	Jan-2030

#### 2.2.1 ZFN related limitations and perspectives

Although ZFNs exhibited their ability to modify a specific gene in mammalian cells, the strategy faces three major limitations. 1) Cannot cut arbitrary gene sequences (Durai, 2005). 2) A ZFN coding gene must be engineered for each specific target site (Porter et al., 2019). 3) The likelihood of off-target gene editing is another drawback of the ZFN technology (Pattanayak et al., 2011). At the beginning, the targeted sequence should contain 5′-GNN, 5′-ANN, 5′-CNN or 5′TNN (Dreier et al., 2001, 2005). Therefore, this technology is costly, laborious, time consuming and requires highly trained researchers for protein engineering (Porter et al., 2019). Recently, the liaison between the ZFP and FokI cleavage domain have been substituted to increase the number of distinct zinc-finger arrays enabling cleavage at a target genomic site (Paschon et al., 2019). In comparison to classical ZFNs, this technique reduced the off-target effect, boosted modification activities, and is more precise. More significantly, it can target and cleave at any intended base (Paschon et al., 2019). In addition, different strategies to engineer the ZFNs and reduce the off-target mutations have also been developed (Ji et al., 2018; Miller et al., 2019a). The assembly of a high specific ZFNs system is highly complex. Researchers isolated naturally occurring ZF modules with different sequence specificities to engineer ZF modules with altered DNA binding specificities (Xiong et al., 2013). Furthermore, several methods are available to increase the specificity and reduced the cellular toxicity of this system by improving the ZFN architecture to develop FokI nuclease domain variants, which could result in a 3,000-fold reduction in off-target indels. (Miller et al., 2007; Miller et al., 2019b).

### 2.3 Transcription activator-like effector nucleases

A class of naturally occurring DNA binding proteins called the Transcription Activator-Like Effector (TALE) has been identified in the plant pathogen Xanthomonas. These TALEs regulate the transcription of several host target genes (Sanjana et al., 2012). TALENs are artificial engineered proteins combining the DNA-binding properties of a TALE protein and the DNA cleavage of the FokI endonuclease (Moscou and Bogdanove, 2009). The central region of TALEs is composed of 34 amino acid repeats amongst which 32 are constant and 2 are variable and recognized as repeat variable diresidues (RVDs) (Scholze and Boch, 2011). RVDs are involved in the DNA target recognition (Boch et al., 2009). Two TALENs target binding sequences are required to form a FokI dimer that induces a DSB (Feng et al., 2014).

TALENs have successfully been used for the modification of T-cell receptors for the treatment of leukemia (Qasim et al., 2017; Benjamin et al., 2020). UCART19, a CAR-T-cell product engineered with TALENs, was tested in children and adults in phase I clinical trials (NCT02808442 and NCT02746952) to cure advanced lymphoid malignancies and refractory B-cell acute lymphoblastic leukemia (B-ALL). These trials demonstrated the potential of UCART19 in patients with aggressive leukemia, but significant adverse events such as cytokine release syndrome, acute graft-versus-host of the skin and infectious complications have also been observed (Benjamin et al., 2020). There are many phase I trials sponsored by Cellectis Inc., using programmed allogenic engineered T-cells expressing different CARs such as UCART123 (NCT04106076, NCT03203369), UCARTCS1A (NCT04142619) and UCART22 (NCT04150497) respectively to treat acute myeloid leukemia, blastic plasmacytoid dendritic cell neoplasm (BPDCN), multiple myeloma and CD22^+^ B cell acute lymphoblastic leukemia. Ongoing clinical trials using this approach are summarized in Table 3.

**TABLE 3:** Summary of TALENs ongoing clinical trials.

Disease	Trial number	Sponsor	Statute	Drug	Phase	Completion date
Myeloma	NCT03190278	Cellectis S.A.	recruiting	UCART123v1.2	I	Oct-2022
Leukemia	NCT04150497	Cellectis S.A.	recruiting	UCART22	I	Oct-2022
Myeloma	NCT04142619	Cellectis S.A.	recruiting	UCARTCS1A	I	Nov-2022

#### 2.3.1 TALENs related limitations and perspectives

Compared to MNs and ZFNs, TALENs exhibits high efficiency, low off-target effects and are proven to target the mitochondrial DNA (Mussolino et al., 2014). Nonetheless, there are some constraints that prevent a more widespread deployment. 1) The repetitive sequences of TALEs make them difficult to construct using polymerase chain reaction (PCR) (Cermak et al., 2011). 2) TALENs are unable to target a methylated DNA, because the methylation of cytosine can potentially abrogate TALE binding and alter recognition by its normal RVD (Deng et al., 2019). Different approaches have been proposed to bypass the challenges associated with the TALEs repetitive sequences are the following: (i) design the ligation-independent cloning techniques (Reyon et al., 2012), (ii) the high-throughput solid-phase assembly (Schmid-Burgk et al., 2013), (iii) the Golden gate cloning (Cermak et al., 2015), and (iv) alternative one-day TALE assembly (Zhang et al., 2020). To improve the efficiency of gene editing, a new bicistronic TALEN termed T2A using classical TALEN coding sequences linked to different reporter molecules by 2A “self-cleaving peptide” has been developed. This improvement could help each TALEN monomer to transcribe from the same reading frame in order to increase the gene editing efficacy. (Mariano et al., 2014; Martín-Fernández et al., 2020). Additionally, Zhang et al. (2017) used deciphered TALEs for 5-hydroxymethylcytosine and 5-methylcytosine to achieve methylation-dependent genome editing and gene activation *in vivo*.

### 2.4 Clustered regularly interspaced short palindromic repeats—CRISPR associated protein 9

CRISPR-Cas9 system is a sophisticated gene editing tool that revolutionized the genome engineering field and generated excitement for the potential of novel therapeutic approaches to treat human diseases. The CRISPR-Cas system is divided into class I and class II (Makarova et al., 2015). The class I uses multi-protein complexes for nucleic acid cleavage and is subdivided into CRISPR-Cas types I, III, and IV. The class II uses a single protein effector domain for the cleavage and is subdivided in CRISPR-Cas type II, V, and VI. The CRISPR-Cas9 belongs to type II system, which is simple to use and thus become the most widely utilized tool for biological research and translational applications (Tang and Fu, 2018).

Over the past decade, CRISPR-Cas9 system has been modified and adapted to become a versatile tool for genome editing in eukaryotes (Ran et al., 2013a; Perez-Pinera et al., 2013; Shalem et al., 2014a; Gao et al., 2019; Casas-Mollano et al., 2020). The system requires a Cas9 nuclease and a single guide RNA (sgRNA) adapted from CRISPR RNA (crRNA), which specifies the target site (the spacer sequence), fused with a trans-activating RNA (tracrRNA), which forms a complex with the crRNA (the scaffold sequence) (Jinek et al., 2012a). The sgRNA forms a stable ribonucleoprotein complex with Cas9 nuclease which initially attaches to a protospacer adjacent motif (PAM) to initiate the first conformational changes of the protein. The targeting activity is driven by 20 nucleotides of RNA-DNA base-pairing between the target DNA strand protospacer and the complementary RNA strand and through interactions between the non-target DNA strand PAM. Subsequently, mediates the second conformational change of the protein which then becomes active. Once activated, the HNH domain of Cas9 cleaves the DNA strand to which the sgRNA is attached and the RuvC domain cleaves at the PAM strand (Jinek et al., 2012b). One of the advantages of CRISPR/Cas9 is that it only requires a sgRNA to specify the DNA sequence where a DSB needs to be generated.

There are many clinical trials based on CRISPR-Cas9 and half of these trials (Phase I and Phase II) have already been successfully completed, the ongoing clinical trials are summarized in Table 4. In a phase I trial (NCT03399448), Pennsylvania University used a multiplex CRISPR-Cas9 to knockout the TCRα, TCRβ, PD-1 genes to treat various malignancies (Stadtmauer et al., 2020). The results showed that the modified T cells engrafted in patients at stable levels for at least 9 months and were barely immunogenic, indicating the feasibility of CRISPR-Cas9 gene editing for cancer immunotherapy (Stadtmauer et al., 2020). In addition to that, some clinical trials moved the technology from ZFNs or TALENs to CRISPR-Cas9 (Yi and Li, 2016; Lu et al., 2020). This was done for example to knockout PD-1 and CD52 for the different types of cancer by electroporating Cas9 and a sgRNA to edit the cells *ex vivo*. The results indicated that the clinical applications of CRISPR-Cas9 gene-edited T-cells are generally safe and feasible (Lu et al., 2020; Frangoul et al., 2021; Modarai et al., 2021). The disruption of erythroid enhancer of the BCL11A gene by CRISPR-Cas9 for the treatment of ß-thalassemia has been observed with serious adverse events. The reported serious adverse events are sepsis and pneumonia in the presence of neutropenia, vaso-occlusive liver disease, abdominal pain and cholelithiasis after CTX001 (autologous CRISPR-Cas9–edited CD34^+^ HSPCs) intravenous (IV) administration, although the substantially raised of hemoglobin levels in fetal blood cells (Frangoul et al., 2021). Two clinical trials are currently conducted to evaluate the safety and efficacy of CRISPR-Cas9 strategies either to restore ß-globin expression in ß-thalassemic patients harboring the IVS-2-654C>T mutation (NCT04205435) or to de-repress γ-globin in transfusion dependent thalassemia (TDT) patients (NCT04211480), but no results are yet available. Recently, another study prepared NTLA-2001, an *in vivo* gene-editing therapeutic agent, made by lipid nanoparticles encapsulating messenger RNA of Cas9 protein and single guide RNA targeting misfolding transthyretin (TTR) responsible of transthyretin amyloidosis. The result showed that the TTR is durably knockout after a single dose of NTLA-2001 (Gillmore et al., 2021; Philippidis, 2021).

**TABLE 4:** Summary of CRISPR ongoing clinical trials.

Disease	Trial number	Sponsor	Drug	Phase	Completion date
Leukemia and lymphoma	NCT03398967	Chinese PLA General Hospital	CD19, CD20, CD22 CAR-T-cells	I/II	May-2022
Leukemia	NCT04557436	Great Ormond Street Hospital for Children NHS Foundation Trust	PBLTT52CAR19	I	June-2022
Gastrointestinal cancer	NCT04426669	Intima Bioscience, Inc.	NA	I/II	Oct-2022
Lymphoma	NCT04767308	Huazhong University of Science and Technology	CT125A	I	Dec-2023
β-thalassemia	NCT04925206	EdiGene (GuangZhou) Inc.	ET-01	I	June 2024
Leber congenital amaurosis	NCT03872479	Editas Medicine, Inc.	EDIT-101	I/II	Mar-2024
β-thalassemia	NCT03655678	Vertex Pharmaceuticals Incorporated	CTX001	II/III	Aug-2024
Sickle cell disease	NCT03745287	Vertex Pharmaceuticals Incorporated	CTX001	II/III	Oct-2024
Transthyretin amyloidosis	NCT04601051	Intellia Therapeutics	NTLA-2001	I	Nov-2024
Leukemia	NCT04037566	Xijing Hospital	XYF19 CAR-T	I	Aug-2025
Myeloid leukemia	NCT05066165	Intellia Therapeutics	NTLA-5001	I/II	Sep-2025
N-H lynphoma	NCT04637763	Caribou Biosciences, Inc.	CB-010	I	Sep-2025
Hereditary angioedema	NCT05120830	Intellia Therapeutics	NTLA-2002	I/II	Dec-2025
Sickle cell disease	NCT04819841	Graphite Bio, Inc.	GPH101	I/II	May-2026
Sickle cell disease	NCT05329649	Vertex Pharmaceuticals Incorporated	CTX001	III	May-2026
Leukemia	NCT04035434	CRISPR Therapeutics AG	CTX110	I	Aug-2026
Sickle cell disease	NCT04774536	Mark Walters, MD	CRISPR_SCD001	I/II	Dec-2026
Myeloma	NCT04244656	CRISPR Therapeutics AG	CTX120	I	Janv-2027
Carcinoma	NCT04438083	CRISPR Therapeutics AG	CTX130	I	Avr-2027
Lymphoma	NCT04502446	CRISPR Therapeutics AG	CTX130	I	May-2027
Sickle cell disease	NCT04208529	Vertex Pharmaceuticals Incorporated	CTX001		Sep-2039

#### 2.4.1 Clustered regularly interspaced short palindromic repeats—CRISPR associated protein 9 limitations and perspectives

The CRISPR-Cas9 technology is associated with some limitation: (i) There are some off-target effects, (ii) the PAM requirement initially limits the editing scope to only sequence near an NGG for canonical SpCas9, and (iii) a more efficient method of delivering the CRISPR/Cas9 system for *in vivo* applications, which includes a protein of about 160 kDa, is required (Woo et al., 2015). Fortunately, the impact of these shortcomings is reduced with the continuous improvement of this technology. Amongst these improvements, Cas9 variants (Kleinstiver et al., 2016a; Slaymaker et al., 2016; Chen et al., 2017; Liu et al., 2020) and engineered sgRNA (Doench et al., 2014; Kocak et al., 2019; Nelson et al., 2022a) have been created to improve specificity and efficiency. Subsequently, the Cas9 variants requiring different PAM sequences which contributes to solve the PAM restriction challenge has also been developed. These variants are described as follows: (i) VQR (D1135V/R1335Q/T1337R) variant which recognizes NGA sequence and (ii) EQR (D1135E/R1335Q/T1337R) variant which recognizes NGA PAM sequence instead of an NGG sequence (Kleinstiver et al., 2015), (iii) SpCas9-NG variant which enables the recognition of NG PAM sequence (Nishimasu et al., 2018), (iv) xCas9 binding to NG, GAT as well as GAA (Hu et al., 2018) and the newest variant (v) SpRY which recognizes the NYN PAM and nearly eliminates the PAM restriction (Walton et al., 2020). These PAM flexibilities significantly increased the genome accessibility. For the delivery part, some CRISPR/Cas9 tools were successfully used to edit cells *in vitro.* This *ex vivo* approach may have certain safety benefits especially regarding off-target gene editing. However, some versions of CRISPR-Cas9 with a sgRNA cannot be efficiently delivered *in vivo* due to their size. Split viruses have been developed to resolve this problem but with reduced expression of the fusion protein. The limitations and optimizations of CRISPR/Cas9 and other limitations such as immunotoxicity, DNA-damage toxicity, etc. have also been detailed. (Tang and Gu, 2020; Uddin et al., 2020; Liu et al., 2021a; Wang et al., 2021b; Khatibi et al., 2021; Maximiano et al., 2021).

## 3 Nickase-based genome engineering technologies

**FIGURE 2:**
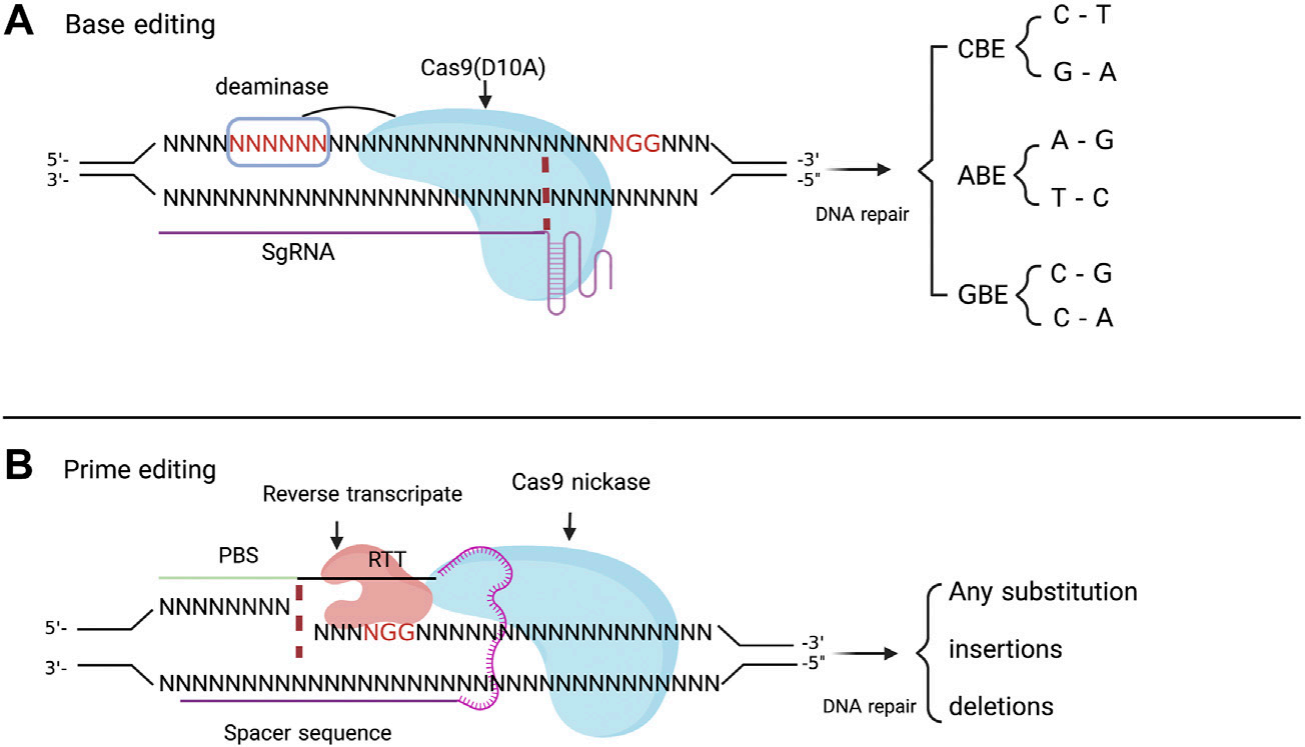
**(A)** represents a basic principle of base editor which is made of sgRNA that target a specific DNA sequence, the Cas9 nickase (D10A) which in interaction with sgRNA binds his recognition domain (PAM sequence) and cleaves the non-PAM DNA strand. The Cas9 is linked to a deaminase which modifies the targeted nucleotide in a window of 5 nucleotides (shown in red color) in the spacer sequence through CBE, ABE, and GBE. The **(B)** depicts the prime editing principle makes of pegRNA which include a spacer sequence, a primer binding site (PBS) and reverse transcript template (RTT), and Cas9 nickase fused with a reverse transcriptase. The pegRNA recognizes the target sequence and provide the desired sequence for modification. Once the Cas9n cleaves the DNA sequence, the reverse transcriptase uses the RTT as template for the synthesis of a new sequence containing the desired edit (gene substitution, insertion and deletion).

DSBs at targeted genomic loci could be associated with serious undesirable effects, including p53 activation (Haapaniemi et al., 2018), translocations (Ghosh et al., 2018), off-target mutation (Vakulskas and Behlke, 2019) and complex undesired products (Paquet et al., 2016). Furthermore, half of all known disease associated gene variants are point mutations (Landrum et al., 2016). Therefore, the Cas9 nickases emerged as useful tools with a targetable property (Cong et al., 2013). The Cas9n D10A and Cas9n H840A are Cas9 variants mediating the cleavage of a single strand of the DNA respectively in the gRNA complementary or non-complementary DNA strand. These Cas9 nickases have been fused with various enzymes, to develop new gene editing technologies, including Base editors [Cytidine base editor (CBE)], Adenosine base editor (ABE) and Prime editor (PE) (Figure 2).

### 3.1 Base editor

Base editors are mainly used to target point mutations that may result in an altered DNA sequence with novel or enhanced functions and gene inactivation (Gaudelli, 2017; Gapinske et al., 2018; Zhang et al., 2019). Briefly DNA base editing requires two main components: a Cas9n fused with a deaminase and a sgRNA which binds to a specific DNA sequence (Komor et al., 2016). Three (3) types of base editors have been created including the Cytidine Base Editor (CBE) (Komor et al., 2016), Adenosine Base Editor (ABE) (Gaudelli, 2017) and Glycosylase Base Editors (GBE) (Zhao et al., 2021a) (Figure 2A).

Theoretically, base editors can function in both dividing and non-dividing cells to address several of single-nucleotide polymorphisms (SNPs) associated with human diseases.

Base editing is being used to study and treat hereditary diseases in a variety of cell types (Zeng et al., 2020; Tekel et al., 2021) and organisms (Kim et al., 2021c), including animal models of human hereditary diseases (Liu et al., 2018; Caso and Davies, 2022). Alternatively, some preclinical experiments demonstrated the prospects of using the base editing technologies to treat diseases. Due to the base editor, hA3A-BE3 creates C-to-T conversion at NGN PAM sites efficiently. Wang et al. (2020) showed that the binding site of BCL11A (TGACCA: −114 to −119) represents an ideal target site for binding with hA3A-BE3 to induce a mutation and raise the level of fetal γ-globin expression to ameliorate the ß-hemoglobinopathies. Furthermore, a transformer BE (tBE) system to eliminate unintended mutations was developed and delivered by AAV into mice creating a premature stop codon in proprotein convertase subtilisin/kexin type 9 (PCSK9) gene which significantly reduced the serum PCSK9 and cholesterol (Wang et al., 2021a). Newby et al. (2021) used mRNA encoding the Base editor to treat hematopoietic stem and progenitor cells (HSPCs) from patient with SCD generating high percentage conversion of the SCD allele (HBB^S^) into Makassar ß-globin (HBB^G^), which is a non-pathogenic variant. It has been shown that base edited CAR T-cells for combinational therapy against T-cell malignancies permitted to enhance molecular remission prior to allo-HSCT for T-cell malignancies (Georgiadis et al., 2021).

#### 3.1.1 BE system limitations and optimizations

The BE system is mainly affected by three aspects: (i) the product purity, (ii) the off-target mutation and 3) the editing window of adjacent sites. Product purity refers to the percentage of edited sequencing reads [reads in which the targeted C has been converted into T but also C to R (G or A)] (Zhang et al., 2018). Furthermore, the product purity is also associated with the frequencies of Indels which are formed during the gene modifying process. Other studies have shown that over-expression of Uracil DNA glycosylase inhibitor (UGI) increased the purity of BE products in human cells (Komor et al., 2017; Jiang et al., 2018; Jang et al., 2021) In addition to UGI, Gehrke et al. (2018) used an engineered human enzyme known as APOBEC3A (eA3A) to develop the eA3A-BE3 base editor which improved target accuracy and reduced bystander mutations. To reduce the Indel formations, the BE4-Gam and CBEmax base editors have been developed (Komor et al., 2017; Huang et al., 2019). The genome targeting function of the base editor caused substantial off-target editing in genomic DNA and RNA (Gaudelli, 2017). Different bioinformatic tools have been developed to overcome the off-target problems including the CRISPR-Cas9/Cpf1 (Kim et al., 2020; Lee et al., 2020), BE-Designer, BE-Analyzer (Hwang and Bae. 2021) and Digenome-seq (Kim et al., 2021b). Furthermore, the engineered deaminases (Rees et al., 2017; Zhou et al., 2019) and gRNA sequences (Kleinstiver et al., 2016b; Hu et al., 2021) have also been built to significantly reduce the off target in the BE system. The range of the editing window for base editing varies according to different application (Kim et al., 2017; Wang et al., 2018b, Wang et al., 2020a; Huang et al., 2019; Tan et al., 2019; Dang et al., 2021; Fu et al., 2021). There are two solutions to set the best range for editing window. 1) When only one specific base pair is required to be changed accurately, the editing window should be minimized to increase the target base accuracy. 2) When CBE system is used to introduce premature stop codons, to produce large-scale saturation mutations, to screen gene function, to locate key amino acid positions in protein domains, etc., a large editing activity window is more advantageous.

### 3.2 Prime editing

Genome editing with base editors effectively induced C→T, G→A, A→G, T→C, C→G, and C→A base substitutions without inducing DSB (Gaudelli, 2017; Zhao et al., 2021b; Kurt et al., 2021). However, they are unable to correct variants beyond these six transition mutations, or other modifications like insertions and deletions of DNA fragments which are successfully achieved by Prime editing (PE). PE uses an engineered Cas9 nickase fused to a reverse transcriptase (RT) enzyme and a modified sgRNA known as prime editing guide RNA (pegRNA) (Figure 2B) (Anzalone et al., 2019). Recent efforts stepwise improved the efficiency of PE system to PEmax system (Chen et al., 2021) and engineer pegRNA known as epegRNA (Nelson et al., 2022b). PE makes possible the accurate insertion up to 1 kb (Wang et al., 2022a) and the deletion of up to 10 kb (Choi et al., 2022) DNA fragment.

As base editing, prime editing has not yet entered clinical trials due to its immature development. However, the potential of PE has so far been demonstrated during the past 2 years *in vitro* (Surun et al., 2020; Habib et al., 2022; Happi Mbakam et al., 2022; Petri et al., 2022; Tremblay et al., 2022) and in animal models (Liu et al., 2021b; Jang et al., 2022; Zheng et al., 2022; Zhi et al., 2022). Unfortunately, the gene modification efficiency is very low in some models. This may be due to the use of the split viruses to overcome the size of PE, which cannot be packed into a single viral delivery vector. Prime editing has the potential to increase the safety and expand the scope of genome-editing in T-cells showing that method is adaptable to enhance the efficiency of CAR T-cell therapy by concurrently introducing additional complex gene edits into T-cells (Petri et al., 2022).

#### 3.2.1 Prime editing limitations and optimizations

The defects of Prime editing are somehow similar to classical CRISPR/Cas9 and base editing as described above, such as the PAM restriction, off-targets and large molecular weight delivery hindering. However, some new progresses have been obtained. Kweon et al. (2021) developed PE2 variants by using various SpCas9 variants named PE2-VQR, PE2-VRQR, PE2-NG, PE2-SpG, and PE2-SpRY. The PE2-SpRY enables targeting 94.4% of pathogenic variants. Furthermore, some studies demonstrated that the PE is not always efficient due to some unknown factors (Li et al., 2020; Lin et al., 2020; Xu et al., 2020). Recently, further research showed that the design of the pegRNA strongly affects the efficiency of prime editors. Researchers demonstrated that optimization of pegRNA sequence widely improved PE efficiency in different cell lines and the efficiency of installing or correcting disease-associated mutations (Jiang et al., 2020, 2022; Kim et al., 2021b; Lin et al., 2021; Nelson et al., 2021).

## 4 Conclusion

During the past decade, gene editing technologies got tremendous improvement in optimizations and applications. With the continuous optimizations of these technologies ZFNs, TALENs and CRISPR-Cas9 have already entered human clinical trials. To date, the majority of clinical applications of these technologies are focused on *ex vivo* gene editing therapeutics. *Ex vivo* editing is highly effective for many medical conditions, such as sickle cell disease, but genome editing should ideally be used for diseases that require *in vivo* cell modification. Having said that, the *in vivo* applications of CRISPR technologies is challenged by issues such as off-target editing, inefficiency, and the stimulation of counterproductive immune responses. Current research addressing these issues may launch new avenues for clinical applications of those nuclease-mediated technologies. Moreover, the novel innovations such as Base editing and Prime editing are still at the pre-clinical stage. All these approaches require DNA strand break that evokes DNA damage responses. Therefore, more efforts are needed to address these limitations including wide off-target events, genome stability, transcription-activation systems and cell proliferation to accelerate the treatment of genetic and infectious diseases.
